# Structural imaging biomarkers of sudden unexpected death in epilepsy

**DOI:** 10.1093/brain/awv233

**Published:** 2015-08-11

**Authors:** Britta Wandschneider, Matthias Koepp, Catherine Scott, Caroline Micallef, Simona Balestrini, Sanjay M. Sisodiya, Maria Thom, Ronald M. Harper, Josemir W. Sander, Sjoerd B. Vos, John S. Duncan, Samden Lhatoo, Beate Diehl

**Affiliations:** 1 1 NIHR University College London Hospitals Biomedical Research Centre, Department of Clinical and Experimental Epilepsy, UCL Institute of Neurology, London WC1N 3BG, UK; 2 2 Epilepsy Society, Chalfont St Peter SL9 0RJ, UK; 3 3 Neuroscience Department, Polytechnic University of Marche, Ancona, Italy; 4 4 The Centre for SUDEP Research, National Institute of Neurological Disorders and Stroke, USA; 5 5 Department of Neurobiology, David Geffen School of Medicine at UCLA, Los Angeles, California, USA; 6 6 Stichting Epilepsie Instellingen Nederland (SEIN), Heemstede, The Netherlands; 7 7 Translational Imaging Group, Centre for Medical Image Computing, University College London, London, UK; 8 8 Epilepsy Centre, Neurological Institute, University Hospitals Case Medical Centre, Cleveland, Ohio, USA

**Keywords:** sudden death, voxel based morphometry, hippocampus, autonomic, sudep risk

## Abstract

Sudden unexpected death in epilepsy is a major cause of premature death in people with epilepsy. We aimed to assess whether structural changes potentially attributable to sudden death pathogenesis were present on magnetic resonance imaging in people who subsequently died of sudden unexpected death in epilepsy. In a retrospective, voxel-based analysis of T_1_ volume scans, we compared grey matter volumes in 12 cases of sudden unexpected death in epilepsy (two definite, 10 probable; eight males), acquired 2 years [median, interquartile range (IQR) 2.8] before death [median (IQR) age at scanning 33.5 (22) years], with 34 people at high risk [age 30.5 (12); 19 males], 19 at low risk [age 30 (7.5); 12 males] of sudden death, and 15 healthy controls [age 37 (16); seven males]. At-risk subjects were defined based on risk factors of sudden unexpected death in epilepsy identified in a recent combined risk factor analysis. We identified increased grey matter volume in the right anterior hippocampus/amygdala and parahippocampus in sudden death cases and people at high risk, when compared to those at low risk and controls. Compared to controls, posterior thalamic grey matter volume, an area mediating oxygen regulation, was reduced in cases of sudden unexpected death in epilepsy and subjects at high risk. The extent of reduction correlated with disease duration in all subjects with epilepsy. Increased amygdalo-hippocampal grey matter volume with right-sided changes is consistent with histo-pathological findings reported in sudden infant death syndrome. We speculate that the right-sided predominance reflects asymmetric central influences on autonomic outflow, contributing to cardiac arrhythmia. Pulvinar damage may impair hypoxia regulation. The imaging findings in sudden unexpected death in epilepsy and people at high risk may be useful as a biomarker for risk-stratification in future studies.

## Abstract (precis)

The mechanisms underlying sudden unexpected death in epilepsy (SUDEP) remain unclear. Wandschneider *et al.* reveal increased amygdalo-hippocampal volume in cases of SUDEP and in individuals at high risk, compared to individuals at low risk and people without epilepsy. Findings are consistent with histopathological reports in sudden infant death syndrome.

## Graphical abstract

**Figure:**
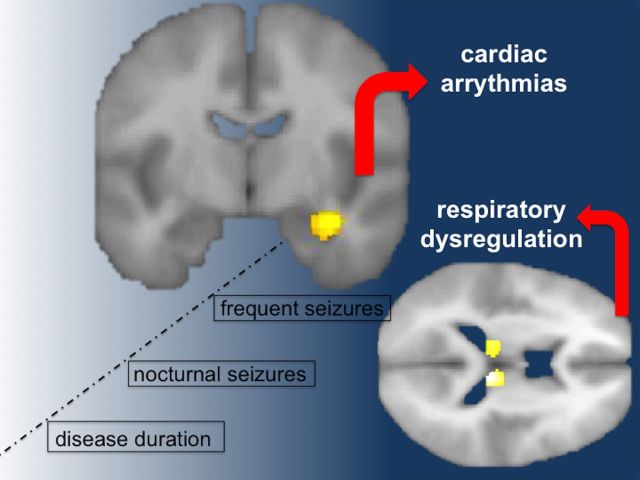
The mechanisms underlying sudden unexpected death in epilepsy (SUDEP) remain unclear. Wandschneider *et al.* reveal increased amygdalo-hippocampal volume in cases of SUDEP and in individuals at high risk, compared to individuals at low risk and people without epilepsy. Findings are consistent with histopathological reports in sudden infant death syndrome.

## Introduction

The incidence of sudden death is 20-fold higher in people with epilepsy than in the general population; sudden unexpected death in epilepsy (SUDEP) is the most common cause of premature death in people with chronic epilepsy. There is currently little understanding of the underlying mechanisms of SUDEP, and post-mortem histopathology has shown no pathognomonic characteristics (Surges *et al.*, 2009; Shorvon and Tomson, 2011; Surges and Sander, 2012). Meta-analyses of SUDEP risk factors (Hesdorffer *et al.*, 2011; Ryvlin *et al.*, 2013*a*) have identified frequent convulsive seizures (≥3/year) as a major risk factor and several studies indicate that unsupervised nocturnal seizures significantly contribute to SUDEP risk (Lamberts *et al.*, 2012). The Mortality in Epilepsy Monitoring Unit Study (MORTEMUS) reported a consistent pattern in video-EEG monitored SUDEP cases, of a convulsive seizure, followed by early and fatal cardiorespiratory dysfunction (Ryvlin *et al.*, 2013*b*). Some studies support a primary respiratory cause with central apnoea, which has been related to postictal generalized EEG suppression, indicating profound depression of CNS functions (Lhatoo *et al.*, 2010). Other studies report primary peri-ictal cardiac arrhythmias and impaired heart rate variability accompanying SUDEP (Surges *et al.*, 2010).

One recent imaging investigation (Mueller *et al.*, 2014) reported severe volume loss in the dorsal mesencephalon in two SUDEP cases. A resting-state functional connectivity study identified reduced functional connectivity between the pons and right thalamus, the midbrain and right thalamus, the anterior cingulate cortex bilaterally and the thalamus, and the right and left thalamus in subjects at high risk compared to those at low risk of SUDEP (Tang *et al.*, 2014). Imaging studies in other conditions with high risk of sudden death have also shown structural changes in brain regions bearing autonomic regulatory or respiratory functions, i.e. the dorsal and ventral medulla, putamen, and bilateral insular cortices in recent-onset obstructive sleep apnoea (Kumar *et al.*, 2014), and the hypothalamus, posterior thalamus, caudal raphe, locus coeruleus, insular cortex and lateral medulla in congenital central hypoventilation syndrome. People suffering from the latter condition are especially at risk for sudden death (Patwari *et al.*, 2010). Neuropathological studies in sudden infant death syndrome (SIDS) report brainstem abnormalities, i.e. brainstem gliosis and defects of neurotransmission in the medulla (Paine *et al.*, 2014). Dentate gyrus abnormalities in the hippocampus were reported in a large subset of 153 sudden infant death syndrome cases, and may reflect defective neuronal migration and proliferation (Kinney *et al.*, 2009, 2015).

Mice carrying mutations in the K_v_1.1 potassium and SCN1A sodium channels have many phenotypes of human SUDEP, e.g. frequent convulsive seizures and premature death. A recent study in these mutant mice demonstrated that cortical EEG suppression coincided with spreading depolarization in the dorsal medulla, a region controlling cardiorespiratory pace making. Depolarizing blockade of these cells prevents normal autoresuscitation and produces cardiorespiratory arrest (Aiba and Noebels, 2015). To elucidate which brain regions may be implicated in SUDEP, we investigated whether regional abnormalities in grey matter volume appear in those who had SUDEP, compared to healthy controls. Due to the low incidence of SUDEP, exploring enriched risk groups has been suggested as a means to increase the yield of future studies (Ryvlin *et al.*, 2013*a*). We explored whether regional imaging findings in people who died of SUDEP can be reproduced in a larger cohort of subjects at high risk for SUDEP. To assess whether imaging findings are common to SUDEP and those at high risk, independent from other epilepsy-related factors, we compared SUDEP cases and those at high risk to a population presumed to be at low risk of SUDEP. We also compared subjects at high risk and low risk of SUDEP to healthy controls.

## Materials and methods

This retrospective study was conducted at a UK tertiary referral centre for epilepsy as part of database research on the ‘Prevention and Risk Identification of SUDEP’, approved by the National Research Ethics Committee (14/SW/0021).

The scans for SUDEP cases, those at low and high risk of SUDEP, and healthy controls were obtained from an overlapping period of case ascertainment, ensuring same imaging protocols were used for acquisition.

Subjects with epilepsy were identified from a general clinical database at the tertiary referral centre. We identified 12 people who died with definite or probable SUDEP, and matched those with 53 living subjects with epilepsy identified from the same database according to the criteria below. All subjects had to have undergone a high-resolution T_1_ volume scan using the identical 3 T MRI scanner as part of their clinical care. Individuals with major brain lesions, such as those after partial temporal lobe resection, were not included to avoid problems with imaging normalization. Sufficient clinical data had to be available to subsequently identify subjects at low or high risk of SUDEP, as described below. All three groups were matched for gender, age, epilepsy syndrome, and epilepsy duration to control for duration-related structural changes. Groups were also matched for lesion pathology where possible.

Healthy controls were comparable to the epilepsy populations for gender and age.

### Characteristics of subjects who died suddenly

Those deceased were classified as probable (*n* = 10) or definite (*n* = 2) SUDEP, according to a recent classification (Nashef *et al.*, 2012). The median age at death was 35.5 [interquartile range (IQR) 2.8] years. Scans were acquired at a median of 2 (IQR 2.8) years antemortem. Videotelemetry data of seizures were available in five SUDEP cases. Further clinical information on the SUDEP cases are shown in Table 1. SUDEP, subjects at high or low risk, as well as control subjects, were comparable for gender and age at scan (Table 2).

**Table 1:** Additional clinical characteristics of the SUDEP cohort

Case	Epilepsy syndrome	SUDEP Category	Lesion on visual inspection of MRI by neuroradiologist	Duration tonic phase (s)	PGES	Duration PGES (s)
1	Juvenile myoclonic epilepsy	Probable	No	N/A	N/A	N/A
2	Focal, left temporal Primary generalized	Probable	Bulky left amygdala with mild FLAIR signal increase	N/A	no	N/A
3	Focal, bitemporal	Probable	no	11	yes	30–43
4	Focal, probably bitemporal	Probable	no	N/A	N/A	N/A
5	Multifocal, left mesial temporal and frontal	Probable	Left hippocampal sclerosis	10	yes	33
6	Focal, frontal	Probable	no	N/A	N/A	N/A
7	Focal, unclassified	Definite	Bilateral periventricular leucomalacia	N/A	N/A	N/A
8	Focal, frontal	Probable	Left hippocampal sclerosis	N/A	yes	5
9	Focal, left hemisphere neocortical	definite	Cavernoma left superior frontal gyrus	6 - 23	no	N/A
10	Unclassified	Probable	Cavernoma right inferior frontal, in white matter	N/A	N/A	N/A
11	Focal, probably bitemporal	Probable	Enlarged left amygdala > hippocampus	N/A	N/A	N/A
12	Focal, left hemisphere	Probable	Right superior temporal DNET	N/A	N/A	N/A

DNET = dysembryoplastic neuroepithelial tumour; FLAIR = fluid-attenuated inversion recovery; N/A =not applicable; PGES = postictal generalized EEG suppression.

**Table 2:** Demographic and clinical parameters

	SUDEP cases (*n = *12)	At high risk (*n = *34)	At low risk (*n = *19)	Controls (*n = *15)	df	*X*^2^	*P*
Age at scan (years) Median (IQR)	33.5 (21.5)	30.5 (12)	30.0 (7.5)	37 (16)	3	2.85	0.241
Age at onset (years) Median (IQR)	16.5 (10)	13.5 (7)	14 (6)	N/A	2	6.21	0.045
Epilepsy duration (years) Median (IQR)	11.5 (24.3)	17 (11.25)	15 (15)	N/A	2	5.74	0.057
Gender, male	8	19	12	7	3	1.42	0.722*
>3 CSs/year	8	24	0	N/A	2	26.09	0.000*
Nocturnal seizures	8	27	0	N/A	2	31.9	0.000*
Polytherapy	4	14	4	N/A	2	2.21	0.347*

Pearson’s chi-square was used for dichotomous variables. As some cells had an expected count < 5, an exact significance test was selected. Kruskal-Wallis test was used for all other variables. CS = convulsive seizure.

*P* < 0.05; asterisk indicates exact *P*.

### Characteristics of people at high or low risk for SUDEP

A risk score was created for each subject according to the most robust epilepsy-specific risk factors for SUDEP identified in recent combined-risk factor analyses (Hesdorffer *et al.*, 2011) that were also implemented in a recent SUDEP imaging study (Tang *et al.*, 2014). Odds ratios for individual SUDEP risk factors were therefore adjusted for different study groups. Those with either nocturnal seizures [odds ratio (OR) = 3.9], or frequent (≥3/year) convulsive seizures (OR = 15.46), were considered ‘high risk.’ Increased SUDEP risk is also associated with young age at disease onset (onset age < 16 years: OR = 1.72), and long disease duration (duration > 15 years: OR = 1.95) (Hesdorffer *et al.*, 2011; Lamberts *et al.*, 2012). For each subject, odds ratios for risk factors were added to define an individual overall risk score. In the SUDEP cohort, 11 of 12 SUDEP cases (91.7%) were correctly identified as high risk subjects if the summed risk score was at least 3.9 (median risk score 19.1, IQR 16.7). One subject with juvenile myoclonic epilepsy had died, probably from SUDEP, but was not known to have suffered from nocturnal seizures or frequent convulsive seizures. A cut-off of 3.9 was therefore used to stratify others into those with high (≥3.9) and low risk (<3.9) of SUDEP.

Individual risk scores and pathology identified on MRI of people at low and high risk for SUDEP are listed in Supplementary Table 1.

SUDEP cases, and those at low and high risk, were matched for epilepsy syndrome (SUDEP: 1/12 generalized genetic epilepsy; high risk: 0/34; low risk: 3/19), and as far as possible for type of pathology (Supplementary Table 2). We were primarily interested in identifying common structures and pathophysiological mechanisms underlying SUDEP and high risk for SUDEP, and the majority of those at low risk (10/19), and high risk (22/34), had no identifiable lesions on a high-resolution 3 T epilepsy protocol clinical MRI brain scan. Videotelemetry data of seizures were available in 30/34 of those at high risk, and 7/19 at low risk. Further information regarding epilepsy classification (as per videotelemetry and/or history) is shown in Supplementary Table 3.

### Controls

Scans of 15 age- and gender-matched healthy control subjects were included from a previous study (Stretton *et al.*, 2013). All controls had normal MRI scans.

### MRI data acquisition

All participants had been previously scanned on the same 3 T GE Signa HDx scanner (General Electric), and were scanned with identical acquisition parameters. We used standard imaging gradients, with a maximum strength of 40 mT/m and slew rate of 150 T/m/s. As part of the clinical sequences, a coronal T_1_-weighted volumetric (3D) scan was acquired with 170 contiguous 1.1-mm thick slices (matrix 256 × 256, in-plane resolution 0.9375 × 0.9375 mm).

### MRI data analysis

We used the Voxel Based Morphometry 8 toolbox (http://dbm.neuro.uni-jena.de/vbm), implemented in Statistical Parametric Mapping (SPM) 8 software (http://www.fil.ion.ucl.ac.uk/spm) for data analysis. Preprocessing included spatial normalization to the Montreal Neurological Institute (MNI) template, segmentation into the different tissue classes (grey matter, white matter, CSF), and modulation to correct for volume changes due to normalization. Intersubject registration was optimized by using the DARTEL (Diffeomorphic Anatomical Registration Through Exponentiated Lie Algebra) algorithm. A quality check implemented in the VBM8 Toolbox did not identify any outliers, and grey matter images were then smoothed with a 10-mm full-width at half-maximum Gaussian kernel (Yasuda *et al.*, 2010).

The smoothed grey matter images were entered into a full-factorial design with group as factor to test for local differences in grey matter volume between groups. Voxels with grey matter values <0.2 (absolute threshold masking) were excluded to avoid edge effects between different tissue types. Age at scan was entered as a nuisance variable into the model.

The statistical threshold was set at *P < *0.001, with a minimum cluster size of 30 contiguous voxels.

### Statistical analysis of demographical and clinical data

Statistical analysis of demographical and clinical data was performed with SPSS Version 20.0 (SPSS Inc.). Pearson’s chi-square test with an exact significance test for cells with a count of less than five was used for dichotomous data. Kruskal-Wallis test was used for all other data (Table 2).

## Results

### Demographic and clinical data

All groups were comparable for gender and age at scan. Epilepsy groups were generally comparable for clinical parameters, except for factors included in the risk scoring, i.e. frequent convulsive seizures, nocturnal seizures, and onset of disease (Table 2). Of the epilepsy groups, 66.7% of SUDEP cases, 35.3% of high risk and 47.3% of low risk had a lesion on the scan (Supplementary Table 2).

### Voxel-based morphometry

**Figure 1:**
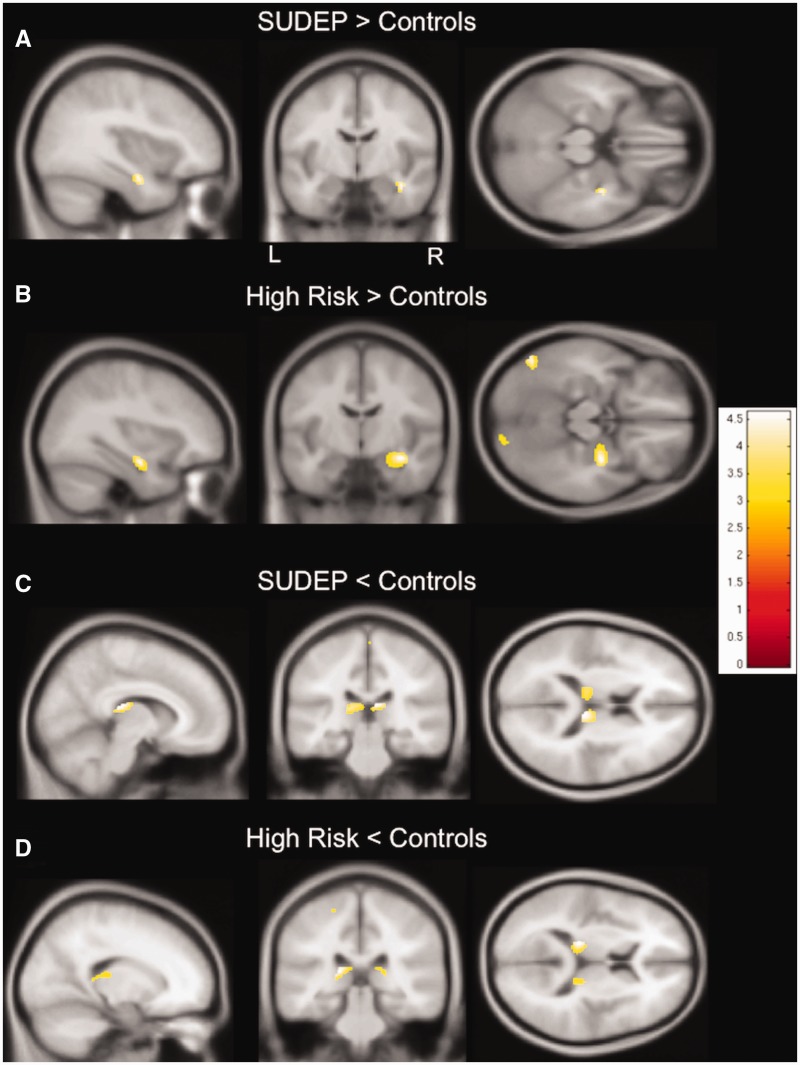
**Regional grey matter volume differences between SUDEP and people at high risk and controls.** (**A**) SUDEP cases show increased grey matter volume in the right hippocampus and parahippocampal gyrus compared to healthy subjects. (**B**) Similarly to SUDEP cases, subjects at high risk show increased grey matter volume in the right hippocampus and parahippocampal gyrus compared to healthy controls. (**C**) Compared to controls, grey matter volume is decreased in SUDEP cases in the pulvinar bilaterally. (**D**) Likewise, grey matter volume is decreased in those at high risk in the left pulvinar, compared to healthy controls. T-values are represented in the coloured bars. *P < *0.001, 30 voxel threshold extent; L = left; R = right.

SUDEP cases showed increased grey matter volume within the right anterior hippocampus, and parahippocampal gyrus (Fig. 1A), and decreased grey matter volume in the pulvinar of the thalamus bilaterally (Fig. 1C), compared to controls. In those at high risk, we found similar changes within these regions, i.e. grey matter volume increase in the right hippocampus and parahippocampal gyrus (Fig. 1B), and decreased grey matter volume in the left pulvinar (Fig. 1C), when compared to controls.

**Figure 2:**
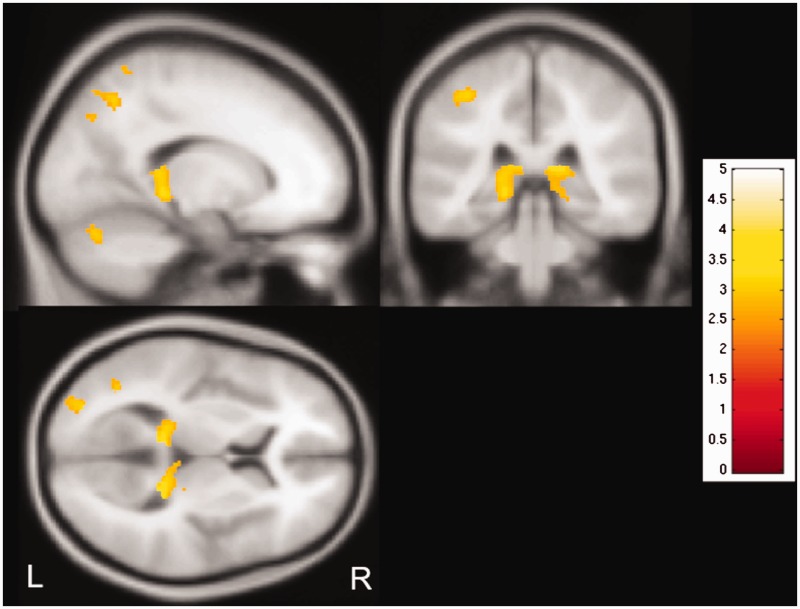
**Correlation of grey matter volume with disease duration.** Regional grey matter volume in bilateral thalamic pulvinar shows a negative correlation with epilepsy duration, i.e. grey matter volume decreases with longer duration (*P < *0.005, 30 voxel threshold extent). T-values are represented in the coloured bar. L = left; R = right.

A *post hoc* analysis across all cases suggested a negative correlation of grey matter volume within the pulvinar bilaterally with disease duration (Fig. 2; *P* < 0.005, 30 voxel threshold extent).

**Figure 3:**
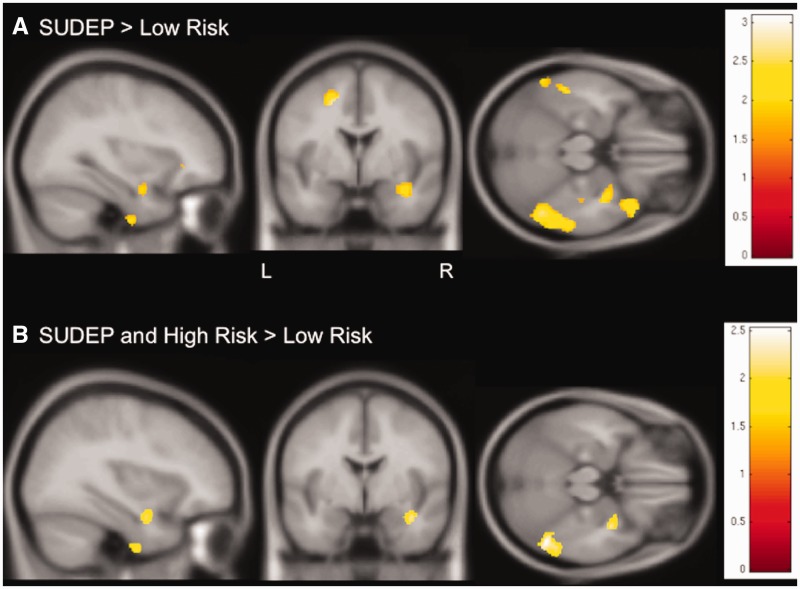
**Regional grey matter volume differences between SUDEP cases and those at high risk in comparison to people at low risk.** (**A**) Similar to findings in comparison to controls (Fig. 1A), but at a lower threshold level (*P < *0.05, 30 voxels threshold extent), SUDEP cases show increased grey matter volume in the right hippocampus and parahippocampal gyrus in comparison to those at low risk. (**B**) People at high risk and SUDEP cases share common areas of increased grey matter volume within the right hippocampus and parahippocampal gyrus when compared to those at low risk (conjunction, *P < *0.05, 30 voxels threshold extent). T-values are represented in the coloured bars. L = left; R = right.

Both SUDEP cases and those at high risk showed areas of increased grey matter volume in the right hippocampus and parahippocampal gyrus, compared to those at low risk (threshold of significance *P < *0.05, 30 voxels threshold extent; Fig. 3).

### Subgroup analyses

**Figure 4:**
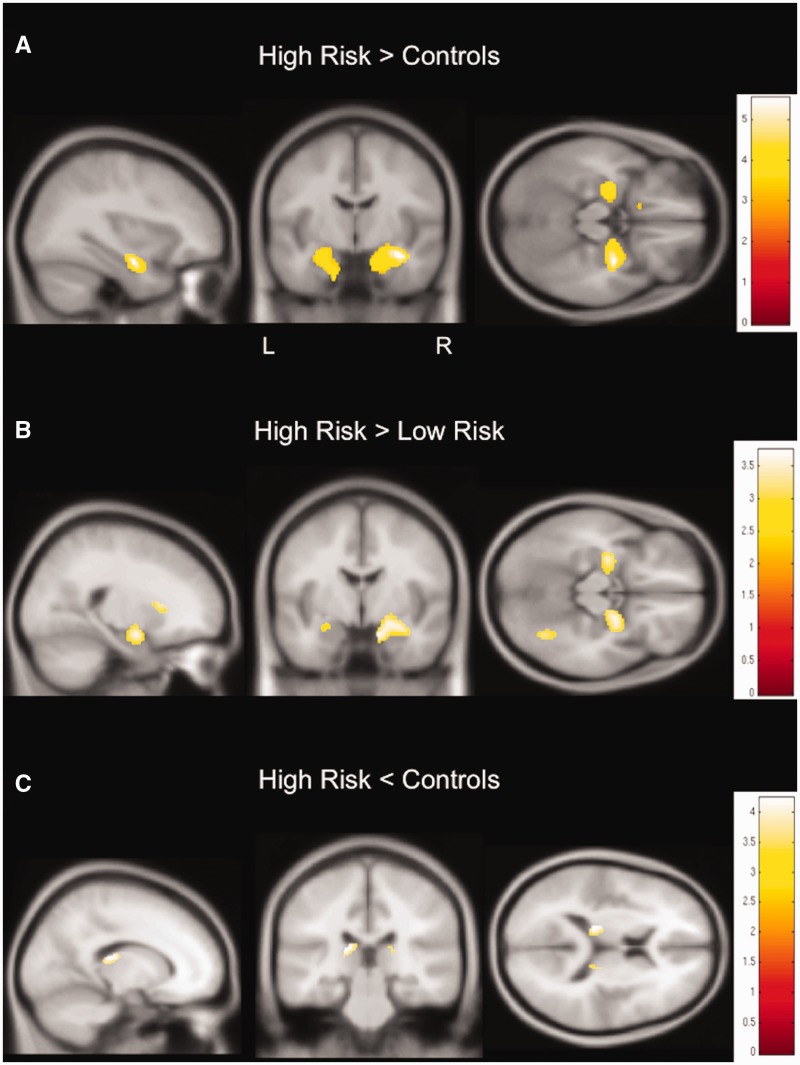
**Regional grey matter volume differences between those at low and high risk with non-lesional epilepsy and controls.** Findings appear similar to previous findings in the whole sample (Fig. 1), but are more bilateral: subjects at high risk without identifiable pathology on clinical structural scans show an increase of grey matter volume in both anterior hippocampi and amygdalae when compared to controls (**A**; *P < *0.001, 30 voxels threshold extent) and when compared to people at low risk (**B**; *P < *0.005, 30 voxels threshold extent). Grey matter volume is decreased in the bilateral posterior thalamus in those at high risk when compared to controls (**C**; *P < *0.005, 30 voxels threshold extent). T-values are represented in the coloured bars.

To ensure that the findings were not driven by gross brain pathologies, we conducted a subgroup analysis in those at risk who had non-lesional MRI scans (low risk *n* = 10; high risk *n* = 22). In the majority of SUDEP cases (66.7%), lesions were evident on clinical scans; hence, due to small sample size, the same subgroup analysis could not be conducted. Age at scan was entered as a nuisance variable. Compared to controls, those at high risk and without lesions still showed increases in anterior hippocampal grey matter volume, as well as in the amygdala, albeit this time bilaterally (Fig. 4A). Similarly, grey matter volume in both hippocampi and amygdalae was increased in people at high risk compared to those at low risk, but more prominent in the right than left amygdala and hippocampus (Fig. 4B).

**Figure 5:**
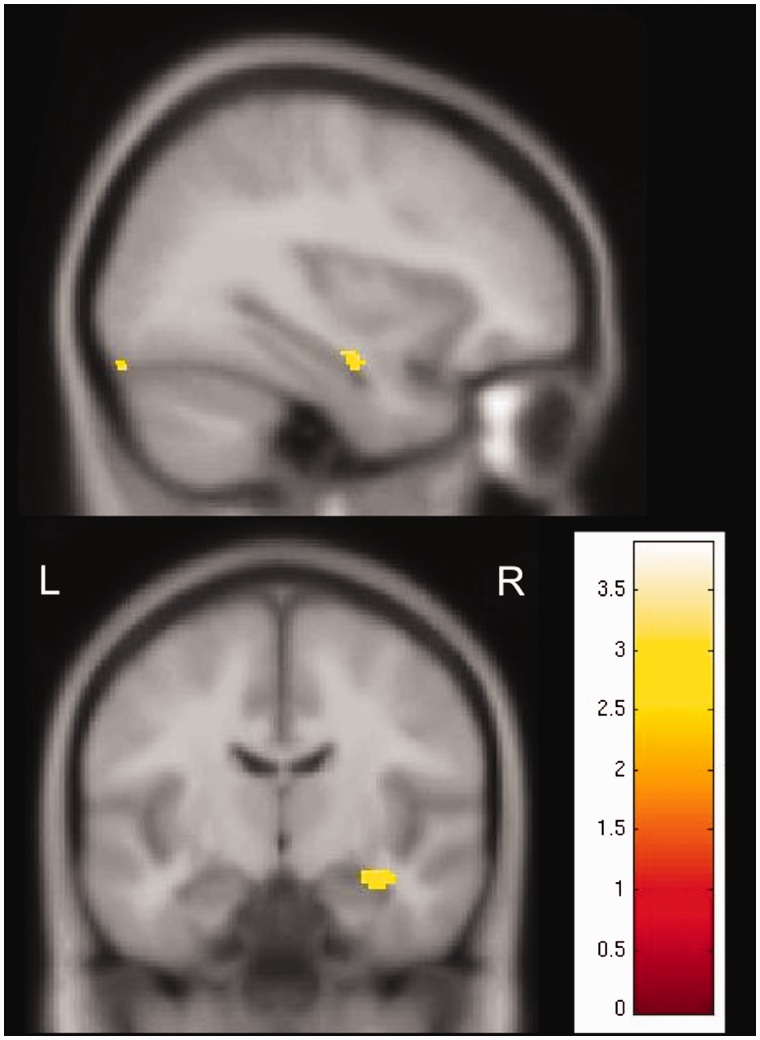
**Common areas of increased grey matter volume in subjects with frequent and less frequent convulsive seizures compared to controls.** Amongst SUDEP cases and people at high risk of SUDEP, subjects with frequent convulsive seizures (i.e. ≥3/year) and less frequent convulsive seizures (<3/year) share common areas of increased grey matter volume in the right hippocampus when compared to healthy controls. Conjunction, *P < *0.005. T-values are represented in the coloured bars. L = left; R = right.

To explore whether findings in the right medial temporal lobe are only related to frequent convulsive seizures, we compared those with more than three convulsive seizures per year to those with fewer convulsive seizures per year in the high risk and SUDEP groups. Fourteen subjects had fewer convulsive seizures (four SUDEP, 10 high risk) and 32 had frequent convulsive seizures (eight SUDEP, 24 high risk). Age at scan and gender were entered as nuisance variables. There were no differences within the medial temporal region between both groups. Compared to controls, both groups showed common areas of increased grey matter volume in the right hippocampus (conjunction, *P* < 0.005; Fig. 5). We also compared total right hippocampal volumes in both groups using an automated segmentation tool (Winston *et al.*, 2013). There were no significant differences in right hippocampal volumes between subjects with frequent and less frequent convulsive seizures (right hippocampal volume in cm^3^ in subjects with less than three convulsive seizures per year: median 3.036, IQR 0.65; in subjects with three or more convulsive seizures per year: median 2.90, IQR 0.52 cm^3^; Mann-Whitney U = 130.000, *P* = 0.646).

**Figure 6:**
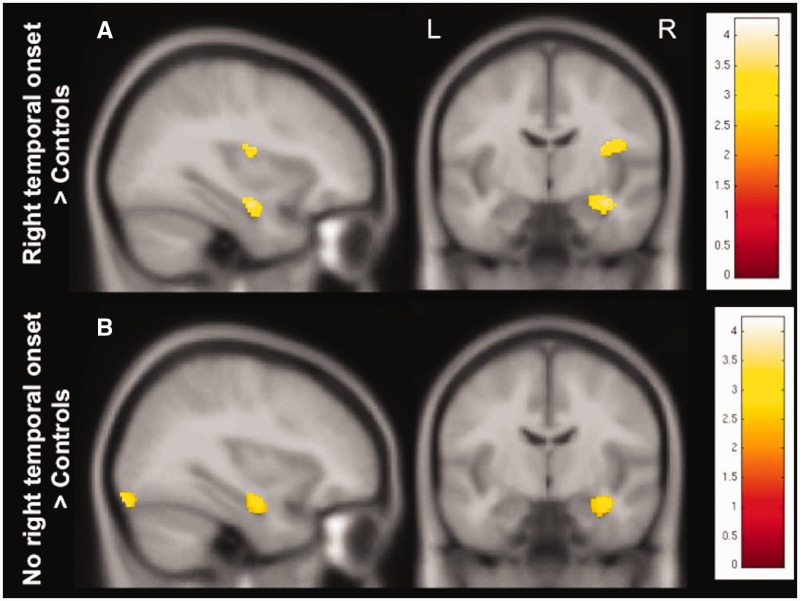
**Grey matter volume changes in subjects with and without right temporal seizure onset.** In comparison to healthy controls, subjects with right temporal seizure onset (Fig. 6A), as well as those without (Fig. 6B) showed an increase of grey matter volume in the right hippocampus (*P < *0.005, 30 voxels threshold extent). T-values are represented in the coloured bars. L = left; R = right.

To relate seizure onset site to right medial temporal findings, subjects with right temporal seizure onset were compared to those with a different, right extratemporal or left hemisphere onset. Ictal EEG data were available in nine SUDEP, 30 high risk and four low risk individuals. In six high risk and one SUDEP case, seizure onset could not be localized and these cases were therefore excluded. There were no differences between these two groups in volumetric findings. In comparison to controls, both groups showed an increase in grey matter volume in the right hippocampus (*P* < 0.005, 30 voxels threshold extent; Fig. 6).

## Discussion

### Anatomical differences between subjects with SUDEP and high risk versus those at low risk

We identified increased grey matter volume within the right hippocampus and parahippocampal gyrus in SUDEP cases and in subjects at high risk for SUDEP, compared to those at low risk and controls. There was increased grey matter volume in both hippocampi, extending to the amygdala when comparing non-lesional high and low risk subjects.

The posterior thalamus (pulvinar) showed disease duration-dependent grey matter volume reduction in all patient groups.

MRIs of all cases and controls were subsequently reviewed again by an experienced neuroradiologist (C.M.), specifically looking for the presence or absence of hippocampal pathology (Table 1 and Supplementary Table 1). No new lesions within these regions were identified on visual inspection of individual cases, suggesting that the findings are at a group level.

Neuropathological studies in sudden unexplained death in childhood and in sudden infant death syndrome have found abnormalities in the same region; dentate gyrus abnormalities in a large subset of 153 sudden infant death syndrome cases (Kinney *et al.*, 2015) were interpreted as a developmental vulnerability, potentially leading to respiratory/autonomic instability, or even autonomic seizures and death during sleep when challenged by homeostatic stressors. Hippocampal and temporal lobe anomalies were also described in 62% of sudden unexplained death in childhood cases (Kinney *et al.*, 2009). Microdysgenetic features of the hippocampal formation included dentate gyrus and subicular anomalies, granular nodular heterotopia, subventricular neuroblasts and hamartia, all indicative of aberrant neurodevelopment. Similar to SUDEP cases, those sudden unexplained death in childhood individuals with structural anomalies were found dead during sleep and in the prone position, and more commonly had an individual or family history of febrile seizures, creating a potential link between hippocampal/temporal lobe maldevelopment, susceptibility to seizures, and sudden death.

Increase in grey matter volume, which has appeared in several epilepsy syndromes in previous voxel-based morphometry studies (Yasuda *et al.*, 2010), has been suggested as indicative of dystopic neurons and diminished grey-white matter demarcation (Yasuda *et al.*, 2010), and findings in the current study may therefore reflect abnormal neurodevelopmental processes. Neuropathological studies in SUDEP show that pathology can be present in the hippocampus (e.g. gliosis) (Zhuo *et al.*, 2012). There are so far, however, no quantitative neuropathological studies of the hippocampus in SUDEP, which would be needed to confirm any subtle abnormalities such as microdysgenesis.

Increased grey matter volumes in the hippocampus may also represent gliosis. Gliosis is a response to injury, and includes neuronal and synaptic functional alterations (Sofroniew, 2009) that have been associated with hyperexcitability in epilepsy (Binder and Steinhäuser, 2006). Gliosis within the hippocampus may therefore alter neuronal activity facilitating the risk of SUDEP, e.g. through hyperexcitability and/or limbic network dysfunction implicating also autonomic function.

A recent study evaluating structural imaging prediction patterns for seizure freedom after surgery in temporal lobe epilepsy found unilateral or bilateral atrophy of the hippocampus, amygdala and entorhinal cortex in most subjects, although one subgroup showed bilaterally increased hippocampal and amygdala volumes (Bernhardt *et al.*, 2015). Subjects in this group were more likely to have unsuccessful epilepsy surgery, supporting the concept that gliosis may facilitate processes of treatment-resistant disease. Histopathology confirmed hippocampal gliosis in almost all subjects of this subgroup. Astrogliosis and cellular hypertrophy have been described in neuropathological studies in hippocampal tissue of subjects with refractory temporal lobe epilepsy (Das *et al.*, 2012). Mild gliosis is also present in cases of amygdala enlargement (Minami *et al.*, 2015).

Longitudinal voxel-based morphometry studies report a decrease of grey matter volume in mesiotemporal structures and the thalamus with longer disease duration and more active disease, i.e. frequent seizures (Bernhardt *et al.*, 2009, 2013; Coan *et al.*, 2009). Similarly, changes in the posterior thalamus correlate with disease duration in our cohort and this suggests a dynamic origin of grey matter volume alterations in our study. A potential mechanism for gliosis in epilepsy could be repeated hypoxic insults, particularly through convulsive seizures (Macey *et al.*, 2009).

That increased grey matter volume may represent gliosis and plasticity following neural injury (Yasuda *et al.*, 2010) is corroborated by data in other sudden death entities: increased grey matter volume in the putamen appears in people with newly-diagnosed obstructive sleep apnoea, who are subjected to repeated hypoxic episodes, with the increased volumes usually attributed to transitional processes in glial death accompanying the neural injury in the syndrome (Kumar *et al.*, 2014).

### Association with autonomic dysfunction and significance of laterality of findings

Several functional imaging studies in humans (Shoemaker *et al.*, 2012, 2015), and stimulation studies in animals (Terreberry and Neafsey, 1987) have identified the hippocampus as an essential component of limbic circuitry modulating autonomic function, with substantial influences on blood pressure regulation (Harper *et al.*, 2000).

Major hippocampal influence on autonomic activity through efferent projections can also be assumed from intracerebral stimulation studies in subjects with refractory epilepsy (Catenoix *et al.*, 2011). These influences are corroborated by reports of people with mesial temporal lobe epilepsy who show decreased heart rate variability in relation to seizures and interictal epileptic discharges, which were more pronounced during sleep, when most cases of SUDEP occur (Moseley *et al.*, 2011). Of interest, heart rate variability normalizes in some after successful temporal lobe epilepsy surgery (Hilz *et al.*, 2002).

Hippocampal grey matter volume increases in our cohort may partially underlie seizure generation and ictal and peri-ictal autonomic dysfunction. However, increased right hippocampal grey matter volume was even present in those individuals with known right medial temporal epilepsy when compared to healthy controls (no cases of hippocampal sclerosis in either group). The increased grey matter volume was surprising, as longitudinal voxel-based morphometry data describe progressive atrophy of the ipsilateral hippocampus in the medial temporal lobe, especially in those subjects with higher seizure frequency and longer epilepsy duration, i.e. higher SUDEP risk (Bernhardt *et al.*, 2009, 2013; Coan *et al.*, 2009). This may suggest that our findings are not associated with a primarily seizure-related autonomic dysfunction; but they may be associated with an interictal autonomic dysregulation. At this time this is a speculative suggestion, and needs investigation of autonomic function in similar cohorts.

### Asymmetry of grey matter volume increases

A significant aspect of the grey matter volume hippocampal increase in SUDEP cases and subjects at high risk was the asymmetry, with the volume changes on the right side. The lateralization of tissue change in an autonomic regulatory area poses a serious concern for sympathetic and parasympathetic outflow. If laterality on sympathetic influences is preserved to medullary output nuclei, the consequences to cardiac arrhythmia generation are severe, as asymmetric sympathetic outflow leads to such phenomena as potentially fatal long Q-T syndrome (Schwartz, 1998). The right insula plays a more prominent role with sympathetic regulation, while parasympathetic regulation is primarily mediated on the left insula (Oppenheimer, 2006), as determined by a series of stimulation, lesion, stroke, and imaging studies, including human epilepsy surgical studies (Oppenheimer *et al.*, 1992). Both injury to the right limbic system and direct insular stimulation have been associated with sympathetic over-regulation (Oppenheimer, 2006). Sudden death after acute right-sided insular strokes and increased complex arrhythmias appears more often than in any other lesion localization (Soros and Hachinski, 2012). Right insular injury in obstructive sleep apnoea shows distorted blood pressure recovery patterns to a challenge (Harper *et al.*, 2003; Henderson *et al.*, 2003) and right hemisphere strokes, particularly when involving the insula, are accompanied by increased nocturnal blood pressure, higher noradrenaline levels and QTc prolongations (Oppenheimer, 2006). The insular effects appear to be mediated by projections to the ventral medial frontal cortex, hypothalamus, and hippocampus through integrated circuitry (Shoemaker *et al.*, 2015). The lateralized (right) increased mesiotemporal grey matter volume in our cohort may contribute to chronic, asymmetric hyper-sympathetic activation, or a sympathetic system lacking in appropriate responsiveness, which would contribute to mechanisms that pose a risk for sudden death.

Similar scenarios develop for obstructive sleep apnoea and for heart failure, which induce severe injury preferentially in the right insula, and consequential very high resting, and unresponsive, sympathetic tone (Macey *et al.*, 2002; Woo *et al.*, 2005). An imbalance between parasympathetic and sympathetic drive places an individual at risk, resulting in a tendency to postictal bradycardia/asystole as noted in the MORTEMUS study (Ryvlin *et al.*, 2013*b*).

### Decreased grey matter volume in the posterior thalamus

A second major finding was that grey matter volume was reduced in the posterior thalamus, and correlated with disease duration. The finding was not unique to SUDEP. A decrease of grey matter volume in the posterior thalamus correlated with disease duration in all subjects with epilepsy (Fig. 2), and one may speculate that those changes may develop in low risk subjects, given sufficient duration of seizures. However, the finding of posterior thalamic grey matter volume should be taken in the context of roles for that structure in respiratory regulation. Substantial evidence, ranging from lesion and stimulation studies in the foetal lamb (Koos *et al.*, 1998, 2004), to functional MRI studies in adolescents and children with congenital central hypoventilation syndrome (Macey *et al.*, 2005), show the significant role of the posterior thalamus in mediating breathing responses following manipulation of oxygen levels, with special participation in the inhibition of breathing following hypoxic exposure (Koos *et al.*, 1998, 2004). We speculate that injury to the posterior thalamus is common in people with epilepsy, that the evidence suggests that disease duration potentiates that injury, and that such injury poses particular risk to the hypoxia normally accompanying ictal episodes, causing thalamic structures to fail to adequately recover from low oxygen. A thalamic role must, however, be viewed in the context that in people who succumbed to SUDEP or who were at high risk also were burdened with right-sided grey matter volume increases in the hippocampal region, which would compromise appropriate blood pressure responses that accompany apnoea. Thus, the combination of injury, diminished posterior thalamic and altered right-sided hippocampal grey matter volume may impose a set of circumstances leading to vital failure.

The mechanisms underlying decreased thalamic grey matter volume should be considered; the decline emerges in several epilepsy syndromes (Yasuda *et al.*, 2010), and appears to be, in part, independent of epilepsy severity, presence of MRI lesions, and duration (Keller *et al.*, 2002). Strong relationships of disease duration and declines in grey matter volume and changes in white matter tract microstructure, i.e. mean fractional anisotropy declines, have been described, and may underlie progressive brain changes in response to active disease, i.e. recurrent seizures (Keller *et al.*, 2012).

High risk for SUDEP has been reported in those who fail epilepsy surgery (Sperling *et al.*, 2005). Persistent seizures in subjects who had undergone amygdalo-hippocampectomy for unilateral temporal lobe epilepsy were associated with preoperative atrophy of bilateral dorsomedial and pulvinar thalamic regions (Keller *et al.*, 2015), with the investigators arguing that these regions are important hubs of seizure modulation and spread. We suggest that the risk may stem from failure of a combined interaction of breathing and blood pressure control.

### Limitations

The criteria used to define our risk groups, and the cut-off between high and low risk, were arbitrary. The finding that SUDEP and those at high risk show similar patterns is consistent with our definition of risk groups. Eleven of 12 SUDEP cases were classified as high risk with our criteria.

A major limitation of our study is to disentangle whether our finding of right hippocampal grey matter volume increase is a specific SUDEP risk factor or rather a marker of severe epilepsy.

As there was only one low risk case in our SUDEP group, we could not establish whether increased right hippocampal grey matter volume is present in SUDEP cases despite being labelled low risk. This would have marked our finding as more SUDEP-specific. *In vivo* imaging biomarkers of SUDEP risk should be present in both subjects who later on died from, and those at high risk of, SUDEP. We argue that the smaller the difference we observe between those two groups, the better our classification and definition of high risk criteria. Similarly, main risk factors for SUDEP—like frequent, uncontrolled convulsive seizures—will have to be present in both SUDEP and high risk groups (Hesdorffer *et al.*, 2011), and hence, are also the major distinguishing factor of high risk versus low risk subjects in our study. By the nature of SUDEP and our study, it is therefore impossible to fully disentangle the effect of severe epilepsy from a specific SUDEP biomarker itself.

Due to methodological challenges (Ashburner and Ridgeway, 2013), there are only few longitudinal voxel-based morphometry studies in people with mesial temporal lobe epilepsy. All of them show grey matter atrophy within mesial temporal structures and beyond (e.g. thalamus) over time, which are more progressive with longer disease duration and higher seizure frequency (Bernhardt *et al.*, 2009, 2013; Coan *et al.*, 2009). Evaluation of subregional mesiotemporal disease progression revealed that progressive atrophy particularly involves the anterior part of the hippocampus (Bernhardt *et al.*, 2013). These reports are in clear contrast with our findings of increased grey matter volume particularly in the anterior hippocampus, and suggest that these are not only caused by frequent seizures. There are poor data on exact seizure counts in our groups, but when subjects in the high risk and SUDEP groups where dichotomized into those with frequent (i.e. more than three convulsive seizures per year) and those with less frequent convulsive seizures, there were no significant group differences within the right hippocampus, but both groups showed common areas of increased right hippocampal grey matter volume when compared to healthy controls (Fig. 5). In addition, total right hippocampal volume measures did not differ between groups. This underscores our argument that the findings represent more specific SUDEP markers than just markers of severe epilepsy.

In keeping with the longitudinal data, posterior thalamic grey matter atrophy correlates with disease duration in our cohort and we can therefore confirm that this finding is not a specific SUDEP biomarker.

We appreciate that epilepsy groups in this study combine various different epilepsy subtypes, and include subjects with lesional and non-lesional MRI scans (Supplementary Table 2). Right hippocampal sclerosis was, however, not present in either epilepsy group, and therefore does not explain differences in right hippocampal grey matter volume. Structural abnormalities were common among our SUDEP population (66.7% of cases), and we acknowledge that our SUDEP group may therefore not be representative of all SUDEP cases.

A previous study (Mueller *et al.*, 2014) described decreased midbrain volume using graph analysis methodology in two SUDEP cases compared to controls. We did not aim to examine brainstem volumes, although our whole-brain analysis included the brainstem; we found no abnormal changes in the brainstem within any group. Voxel-based morphometry has substantial limitations in evaluating brainstem segmentation, due to the difficulty in resolving internal brainstem architecture reliably and consistently (Lambert *et al.*, 2013). Disturbances in brainstem attributes may be better evaluated with newer procedures for examining tissue changes, such as diffusion MRI.

## Conclusion

Increased right hippocampal and parahippocampal grey matter volume and grey matter volume decline in the posterior thalamus appear to be related to SUDEP risk. In the case of grey matter volume increases, the relationship is independent of markers of severe epilepsy, such as frequent convulsive seizures. The volume increases are potentially of dynamic origin, representing gliosis in response to repetitive injury from severe epilepsy, while the thalamic volume declines may result from excitotoxic or other injury sources. The thalamic injury may lead to an inability to recover breathing to a hypoxic challenge from apnoea, while the hippocampal/parahippocampal pathology may contribute to asymmetric influences on autonomic outflow, establishing circumstances for cardiac arrhythmia and hypotension. The structural changes may be useful biomarkers to assist determination of pathophysiology of SUDEP.

## Funding

This work was undertaken at UCLH/UCL who receives a proportion of funding from the Department of Health's NIHR Biomedical Research Centres funding scheme. It was partially funded by the NIH –National Institute of Neurological Disorders and Stroke (U01-NS090405-01 and U01-NS090407-01. The Center for SUDEP Research) and Wellcome Trust (project grant 083148). S.B.V. is funded by the National Institute for Health Research University College London Hospitals Biomedical Research Centre (NIHR BRC UCLH/UCL High Impact Initiative). J.W.S. is supported by the Marvin Weil Epilepsy Research Fund.

## Supplementary material

Supplementary material is available at *Brain* online.

Supplementary Table 1

## Abbreviation

SUDEP: sudden unexpected death in epilepsy
