# Supplementary material for: Structural imaging biomarkers of sudden unexpected death in epilepsy
Source: Brain. 2015 Aug 11;138(10):2907–19. doi: 10.1093/brain/awv233 (PMC4671481; doi:10.1093/brain/awv233)
Supplement: Supplementary Table 1 [file 886333c04f5c553fadefd3673d49fe61_brain-2015-00721-File011.pdf]

Supplementary Table 1. Additive odds ratios and individual pathology demonstrated on MRI

**Subject**	**group**	**total risk score**	**early onset**	**long duration**	**CS >3/year**	**nocturnal seizures**	**lesion on MRI**
**1**	Low risk	0	0	0	0	0	right temporal occipital FCD
**2**	Low risk	1.95	0	1.95	0	0	no lesion
**3**	Low risk	1.95	0	1.95	0	0	no lesion
**4**	Low risk	3.67	1.72	1.95	0	0	left superior temporal gyrus non specific focus
**5**	Low risk	3.67	1.72	1.95	0	0	no lesion
**6**	Low risk	0	0	0	0	0	no lesion
**7**	Low risk	3.67	1.72	1.95	0	0	right inferior parietal cystic lesion (likely DNET)
**8**	Low risk	1.95	0	1.95	0	0	no lesion
**9**	Low risk	1.72	1.72	0	0	0	no lesion
**10**	Low risk	1.72	1.72	0	0	0	no lesion
**11**	Low risk	0	0	0	0	0	no lesion
**12**	Low risk	0	0	0	0	0	no lesion
**13**	Low risk	0	0	0	0	0	right anterior temporal/amygdala cavernous haemangioma

**14**	Low risk	3.67	1.72	1.95	0	0	no lesion
**15**	Low risk	3.67	1.72	1.95	0	0	hypoxic injury
**16**	Low risk	0	0	0	0	0	exophytic cavernoma or lipoma left inferior colliculus
**17**	Low risk	1.72	1.72	0	0	0	left hippocampal cavernoma or DNET/left hippocampal sclerosis
**18**	Low risk	3.67	1.72	1.95	0	0	right fusiform carvernoma
**19**	Low risk	1.72	1.72	0	0	0	left hippocampal sclerosis
**20**	High risk	21.08	1.72	0	15.46	3.9	right hippocampal sclerosis
**21**	High risk	23.03	1.72	1.95	15.46	3.9	no lesion
**22**	High risk	5.62	1.72	0	0	3.9	left hippocampal sclerosis
**23**	High risk	7.57	1.72	1.95	0	3.9	left precentral DNET
**24**	High risk	15.46	0	0	15.46	0	no lesion
**25**	High risk	19.13	1.72	1.95	15.46	0	subtle left insular malformation
**26**	High risk	3.9	0	0	0	3.9	no lesion

**27**	High risk	19.13	1.72	1.95	15.46	0	right parietal damage or dysplasia
**28**	High risk	19.36	0	0	15.46	3.9	no lesion
**29**	High risk	21.08	1.72	0	15.46	3.9	left temporal dysplasia, small left hippocampus
**30**	High risk	7.57	1.72	1.95	0	3.9	no lesion
**31**	High risk	5.62	1.72	0	0	3.9	no lesion
**32**	High risk	19.36	0	0	15.46	3.9	left hippocampal sclerosis
**33**	High risk	7.57	1.72	1.95	0	3.9	right inferior parietal cortical dysplasia
**34**	High risk	23.03	1.72	1.95	15.46	3.9	no lesion
**35**	High risk	3.9	0	0	0	3.9	no lesion
**36**	High risk	21.31	0	1.95	15.46	3.9	left frontal non specific white matter focus
**37**	High risk	7.57	1.72	1.95	0	3.9	no lesion
**38**	High risk	19.36	0	0	15.46	3.9	right superior temporal gyrus/polar haematoma (old)
**39**	High risk	23.03	1.72	1.95	15.46	3.9	cerebellar atrophy
**40**	High risk	5.62	1.72	0	0	3.9	no lesion
**41**	High risk	19.13	1.72	1.95	15.46	0	no lesion

**42**	High risk	23.03	1.72	1.95	15.46	3.9	mature damage left>right gyrus rectus
**43**	High risk	21.31	0	1.95	15.46	3.9	no lesion
**44**	High risk	15.46	0	0	15.46	0	no lesion
**45**	High risk	7.57	1.72	1.95	0	3.9	hypothalamic hamartoma
**46**	High risk	23.03	1.72	1.95	15.46	3.9	no lesion
**47**	High risk	19.13	1.72	1.95	15.46	0	no lesion
**48**	High risk	19.13	1.72	1.95	15.46	0	no lesion
**49**	High risk	21.08	1.72	0	15.46	3.9	no lesion
**50**	High risk	23.03	1.72	1.95	15.46	3.9	no lesion
**51**	High risk	23.03	1.72	1.95	15.46	3.9	no lesion
**52**	High risk	21.08	1.72	0	15.46	3.9	left supramarginal gyrus dysplasia
**53**	High risk	23.03	1.72	1.95	15.46	3.9	no lesion
**54**	SUDEP	0	0	0	0	0	no lesion
**55**	SUDEP	23.03	1.72	1.95	15.46	3.9	bulky left amygdala with mild FLAIR signal increase
**56**	SUDEP	19.36	0	0	15.46	3.9	no lesion
**57**	SUDEP	15.45	0	0	15.46	0	no lesion
**58**	SUDEP	19.13	1.72	1.95	15.46	0	left hippocampal sclerosis
**59**	SUDEP	5.85	0	1.95	0	3.9	no lesion

**60**	SUDEP	21.08	1.72	0	15.46	3.9	bilateral periventricular leucomalacia
**61**	SUDEP	23.03	1.72	1.95	15.46	3.9	left hippocampal sclerosis
**62**	SUDEP	3.9	0	0	0	3.9	cavernoma left superior frontal gyrus
**63**	SUDEP	21.08	1.72	0	15.46	3.9	cavernoma right inferior frontal, in white matter
**64**	SUDEP	19.13	1.72	1.95	15.46	0	enlarged left amygdala > hippocampus
**65**	SUDEP	3.9	0	0	0	3.9	right superior temporal DNET

DNET = dysembryoplastic neuroepithelial tumor; CS = convulsive seizures; FCD = focal cortical dysplasia; SUDEP = sudden unexpected death in epilepsy

	Low risk<br>N = 19	High risk<br>N = 34
No lesion	10	22
Hippocampal sclerosis<br>Left/Right	 1/0	 2/0
Focal cortical dysplasia<br>Left/Right	3<br>1/2<br>- 1 left/ 1 right temporo-occipital<br>- 1 right parieto-occipital	6<br>4/2<br>- 1 left insular<br>- 1 left medial temporal<br>- 1 right supramarginal gyrus<br>- 1 entire left temporal lobe<br>- 1 left inferior parietal<br>- 1 right anterior temporal
Cavernoma<br>Left/Right	2<br>0/2<br>- right temporal pole<br>- right fusiform gyrus	1<br>0/1<br>- right temporal pole
DNET<br>Left/Right	1<br>0/1<br>- left amygdala	0
Hamartoma	0	1<br>- hypothalamic

Ischaemic lesions<br>Left/Right	1<br><br>- perinatal, leading to ventriculomegaly	1<br>0/1<br>- right parieto-occipital
Unclassified lesions<br>Left/Right	1<br>1/0<br>- left frontal	1<br>1/0<br>- left superior frontal
Lateralization<br>Left/Right	3/5	7/3

Supplementary Table 2. Structural abnormalities

DNET = dysembryoplastic neuroepithelial tumor

	Low Risk<br>N = 19	High risk<br>N = 34
Videotelemetry data available	7	30
Epilepsy syndrome		
temporal (left/right/bitemporal)	4 (1/1/2)	6 (3/2/1)
temporo-occipital (L/R/non-lateralizing)	2 (0/2/0)	1 (0/0/1)
fronto-temporal (L/R/bilateral)	0	6 (4/1/1)
frontal (L/R/non-lateralizing)	0	6 (1/2/3)
parieto-occipital (L/R)	3 (1/2)	3 (1/2)
hemisphere (L/R)	0	1 (1/0)
Lateralisation	2 left / 5 right / 11 non-lateralized	10 left / 7 right / 13 non-lateralized
Focal, non-localisable	6	6
Idiopathic generalised	3	0

Supplementary Table 3. Epilepsy classification in the at risk populations

L = left; R = right
