# Supplementary figures and images for: Structural imaging biomarkers of sudden unexpected death in epilepsy
Source: Brain. 2015 Aug 11;138(10):2907–19. doi: 10.1093/brain/awv233 (PMC4671481; doi:10.1093/brain/awv233)

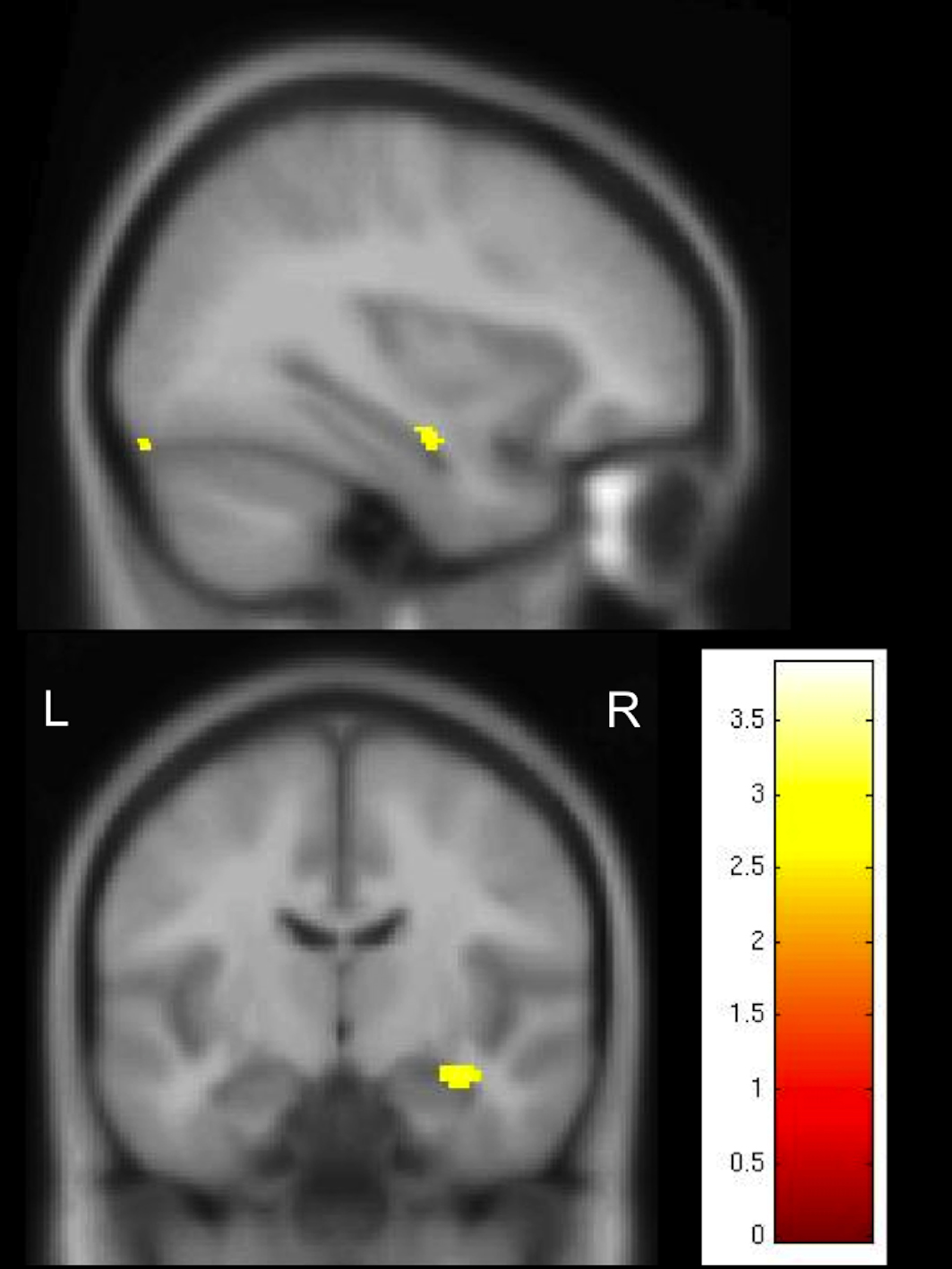

Supplement: Supplementary Table 1 [file d3e304ab9b4abb9be0fd74ca347a4601_brain-2015-00721-File012.jpg]
